# Massive perineal defect after radio‐chemotherapy

**DOI:** 10.1002/ccr3.824

**Published:** 2017-01-27

**Authors:** Maddalena Zippi, Sara Ramponi, Carla Narduzzi, Antonio Maria Alberti

**Affiliations:** ^1^ Unit of Gastroenterology and Digestive EndoscopySandro Pertini HospitalRomeItaly; ^2^ Unit of OncologySandro Pertini HospitalRomeItaly

**Keywords:** Intensity‐modulated radiotherapy, posterior perineum, radio‐chemotherapy, surgery

## Abstract

Herein, we present a rare complication of radio‐chemotherapy. A young man presented with loss of urine from the posterior perineum that showed a massive disappearance. He underwent Hartmann procedure for rectal neoplasia, afterwards treated with intensity‐modulated radiotherapy (IMRT) (until 67 Gy) and chemotherapy with FOLFOX protocol (Leucoverin calcium, Fluorouracil, Oxaliplatin).

## What is This Condition and How Should It Be Treated?

**Figure 1:**
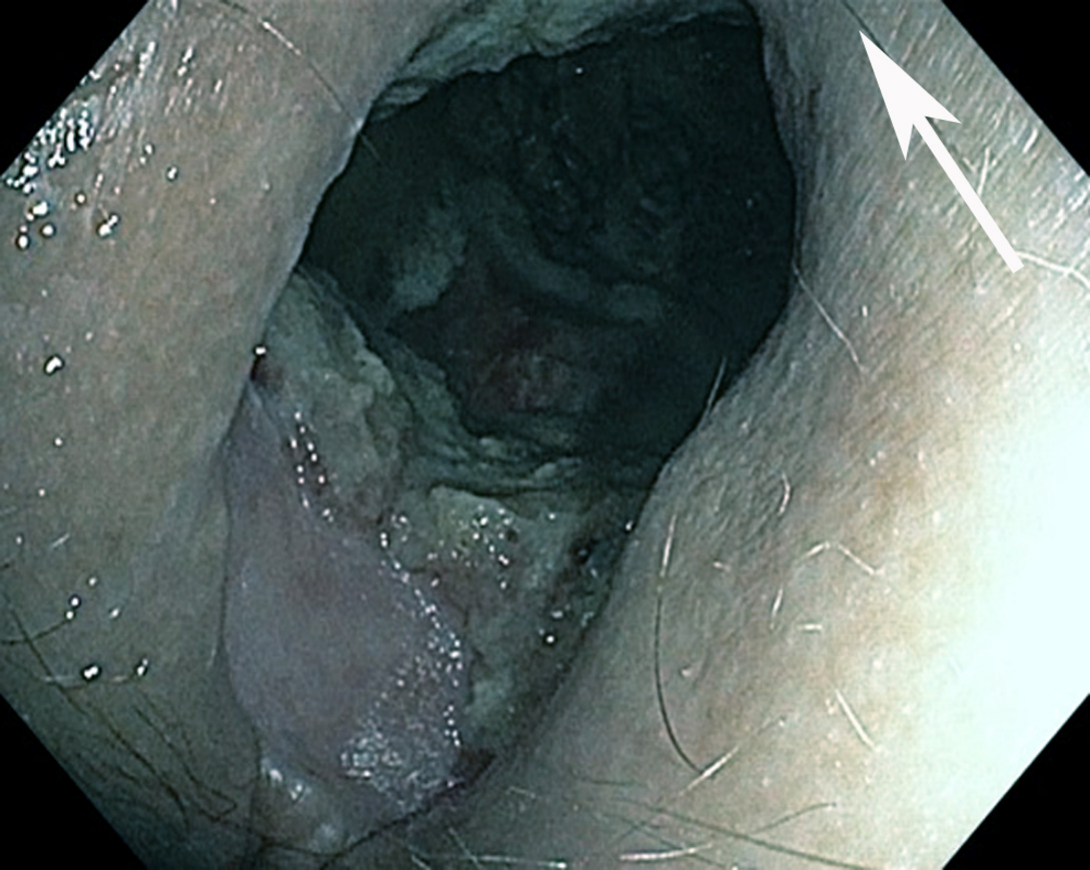
Perianal tissue injury (presacral space, white arrow).

A 40‐year‐old man underwent Hartmann procedure for the treatment of rectal cancer. Subsequently, he was treated with six cycles of intensity‐modulated radiotherapy (maximum dose of 67 Gy) and 12 sessions of adjuvant chemotherapy with FOLFOX protocol (leucovorin calcium, fluorouracil, oxaliplatin). After 3 years, he presented with loss of urine from the posterior perineum. Physical examination showed a complete disappearance of the posterior portion of the perineum, including muscle–skin structures, with signs of ulceration (Fig. 1). Injection of methylene blue into the bladder catheter under endoscopic vision demonstrated a continuous lesion on the posterior bladder wall. The patient was treated conservatively with placement of bilateral percutaneous kidney drainage followed by surgical suture of the lesion. Reconstruction procedure was proposed to him, but he declined to undergo additional surgeries. It is known that complications related to radio‐chemotherapy for rectal cancer, especially after surgical resection, vary from patient to patient. In rare cases, it is possible to carry out a complete reconstruction of the extended chronic presacral defect, which has not been possible in our case 1.

## Authorship

MZ, SR, CR, AMA: All authors have participated in the work: (1) substantial contributions to conception and design, acquisition of data, or analysis and interpretation of data; (2) drafting the article or revising it critically for important intellectual content; and (3) final approval of the version to be published.

## Conflict of Interest

None declared.

## Informed Consent

Written informed consent was obtained from the patient before the procedure.
